# One-Pot Formation of Pairing Proto-RNA Nucleotides and Their Supramolecular Assemblies

**DOI:** 10.3390/life13112200

**Published:** 2023-11-12

**Authors:** Tyler P. Roche, Pranav J. Nedumpurath, Suneesh C. Karunakaran, Gary B. Schuster, Nicholas V. Hud

**Affiliations:** School of Chemistry & Biochemistry, Georgia Institute of Technology, Atlanta, GA 30332, USA; tyler.roche@gmail.com (T.P.R.); pnedumpurath3@gatech.edu (P.J.N.); suneesh.karunakaran@chemistry.gatech.edu (S.C.K.); schuster@gatech.edu (G.B.S.)

**Keywords:** RNA World, origin of life, prebiotic, proto-nucleotide, pre-RNA

## Abstract

Most contemporary theories for the chemical origins of life include the prebiotic synthesis of informational polymers, including strong interpretations of the RNA World hypothesis. Existing challenges to the prebiotic emergence of RNA have encouraged exploration of the possibility that RNA was preceded by an ancestral informational polymer, or proto-RNA, that formed more easily on the early Earth. We have proposed that the proto-nucleobases of proto-RNA would have readily formed glycosides with ribose and that these proto-nucleosides would have formed base pairs as monomers in aqueous solution, two properties not exhibited by the extant nucleosides or nucleotides. Here we demonstrate that putative proto-nucleotides of the model proto-nucleobases barbituric acid and melamine can be formed in the same one-pot reaction with ribose-5-phosphate. Additionally, the proto-nucleotides formed in these reactions spontaneously form assemblies that are consistent with the presence of Watson–Crick-like base pairs. Together, these results provide further support for the possibility that heterocycles closely related to the extant bases of RNA facilitated the prebiotic emergence of RNA-like molecules, which were eventually replaced by RNA over the course of chemical and biological evolution.

## 1. Introduction

The ability of RNA to function as an informational polymer and to catalyze reactions is seen by many as support for the hypothesis that RNA was the primary polymer of early life before the emergence of DNA and coded protein synthesis (the RNA World hypothesis) [1,2]. A “strong” interpretation of the RNA World hypothesis imagines that RNA was the first polymer of life [3]. This view has fueled efforts to demonstrate plausible prebiotic syntheses for the *canonical* nucleosides and nucleotides—the ribonucleotides of coding RNA in extant life [4]. In a recent report, Carell and co-workers described significant progress toward this objective, describing the first concurrent, abiotic synthesis of purine- and pyrimidine-containing nucleosides [5]. However, significant challenges remain concerning the RNA World hypothesis. Among these is the observation that the canonical nucleobases, as free bases or as mononucleotides, do not spontaneously pair in aqueous solution under reasonable prebiotic conditions (a conundrum that we have called the *Paradox of Base Pairing* [6]). This fact, and the unmet challenge of the abiotic formation of phosphodiester bonds between nucleotides, has led us and others to postulate that RNA was not the first genetic polymer of life, but rather a latter member of an evolutionary line of genetic polymers [7]. In this context, it is hypothesized that the earliest ancestor of RNA, or proto-RNA, contained *noncanonical* nucleotides (e.g., with different bases) that were replaced sequentially over the course of chemical and biological evolution. The concept of proto-RNA and pre-RNAs (genetic polymers intermediate between proto-RNA and RNA) has been the subject of experimental investigation during the past decade, including efforts to understand which alternative backbones, linker moieties, and recognition units can exhibit RNA-like properties (i.e., double helix formation and catalytic activity) [8]. Modification of the RNA backbone often dramatically changes its properties, for example, causing the stability of the double helix to be highly sequence-dependent [9]. In contrast, substitution of the canonical bases by alternative heterocycles that closely preserve the geometry of the paired bases can provide RNA-like polymers with duplex stabilities similar to those of extant nucleic acids [10].

**Figure 1:**
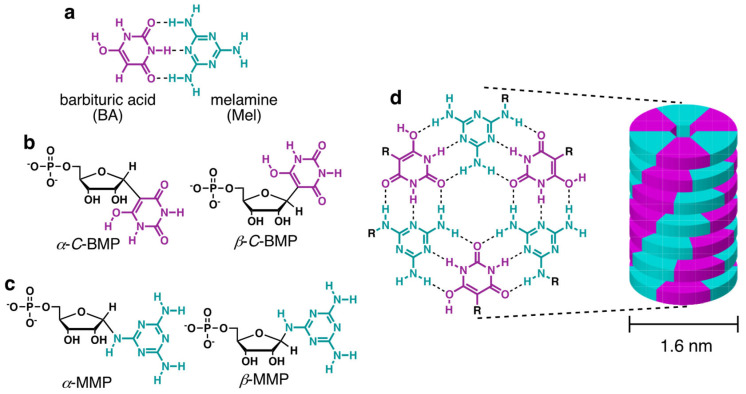
Structures of molecules and assemblies discussed in the text. (**a**) Structures of barbituric acid (BA) and melamine (Mel) shown as hydrogen bonded in a base pair that is similar in geometry to the canonical Watson–Crick base pairs. (**b**) Structures of the *α* and *β* isomers of the BA *C*-nucleotide (barbituric acid monophosphate: BMP) formed by the reaction of BA with ribose-5-phosphate. (**c**) Structures of the *α* and *β* isomers of the Mel nucleotide (melamine monophosphate: MMP) formed by the reaction of Mel with ribose-5-phosphate. (**d**) Structure of the hexad formed by the pairing of three BA and three Mel bases/nucleotides, and an illustration of the linear assembly formed by the stacking of BA-Mel hexads in water.

Simply enumerating the chemical space defined by the pyrimidine, purine, and *s*-triazine heterocycles with H, NH_2_, and O as exocyclic groups—the class of molecules that potentially existed on the prebiotic Earth along with the canonical nucleobases—reveals 91 molecules from which life could have selected the first base-pairing compounds for a proto-RNA [11]. From these possibilities, previous investigations revealed that barbituric acid (BA), melamine (Mel), and 2,4,6-triaminopyrimidine (TAP) (hereinafter referred to generically as “bases”) react with ribose, ribose-5-phosphate (R5P), and other aldose sugars in aqueous solutions to form nucleosides and nucleotides [12,13,14,15] (Figure 1a–c). These putative proto-nucleobases are of further prebiotic interest because both have been synthesized in the same model prebiotic reaction [16], both have good photostability (minimal UV absorbance above 210 nm) [17,18], and both have the ability to assemble as monomers in aqueous solution [19]. The assemblies formed by these bases and their derivatives contain hydrogen-bonded hexameric structures with Watson–Crick-like base pairs (Figure 1a,d). The large hydrophobic surface area of these hexads (ca. 2 nm^2^) drives their stacking in water (Figure 1d), which results in the formation of long, stiff, helical supramolecular polymers that are observable by atomic force microscopy (AFM) and electron microscopy and have been further characterized by X-ray fiber diffraction [12,13].

Our previously reported model prebiotic syntheses of BMP and MMP (nucleotides of BA and Mel, respectively, Figure 1b,c) were carried out in separate reactions with different conditions due to the different p*K*_a_ values of these two heterocycles [13]. This requirement might have necessitated their independent syntheses being separated in time or space, which introduces a level of complexity to their prebiotic formation. Now, in parallel with Carell’s report, here we describe the successful one-pot formation of BMP and MMP under exceptionally mild conditions. Furthermore, in contrast to the canonical nucleobases, we observe spontaneous, selective base-pairing of these proto-nucleotides even in the crude mixture of reaction products.

## 2. Materials and Methods

### 2.1. Chemicals

Barbituric acid (BA), melamine (Mel), and d-ribose-5-phosphate (R5P) were purchased from Sigma-Aldrich and used as received.

### 2.2. Preparation of Experimental Mixtures

Mixtures of R5P with BA and Mel were prepared by combining stock solutions of R5P, BA, and Mel in deionized water to create two reaction mixtures: a dilute mixture containing 20 mM R5P, 10 mM BA, and 10 mM Mel, and a concentrated mixture containing 100 mM R5P, 50 mM BA, and 50 mM Mel. These mixtures were subjected to the reaction conditions described below.

### 2.3. Analysis

At the conclusion of each reaction, the crude product solution was frozen to stop further changes. Each product mixture was subsequently analyzed by NMR and CD spectroscopies and by AFM. Samples for NMR spectroscopy were prepared by freeze-drying portions of the crude reaction mixture, followed by dissolution in D_2_O containing a quantitatively known small amount of sodium trimethylsilylpropanesulfonate, DSS (a ^1^H chemical shift standard). These samples were analyzed using a Bruker Avance IIIHD 800 MHz NMR using standard 1D ^1^H methods. CD analysis was performed by taking a 20 µL aliquot of the intermediate (suspension) phase of the thawed crude reaction mixture and adding 5 µL of 5 M NaCl before placing between the two plates of a strain-free 0.01 mm quartz demountable cell (Starna Cells, Inc., Atascadero, CA, USA), which were analyzed using a JASCO 810 CD spectropolarimeter with 3 spectra collected and averaged per temperature, and with sample temperature increased in increments of 4 °C. AFM images were obtained with a Nanoscope IIIa (Digital Instruments) in tapping mode using Si tips (Vistaprobes, 48 N/m) on freshly cleaved mica. A 2 μL sample of the assembly solution, prepared the same as for CD, was spread over the mica using N_2_ flow and dried with N_2_ gas. Excess sample was washed with cold water, and the mica surface was dried by N_2_ flow.

## 3. Results

### One-Pot Formation of Nucleotides

An initial survey of the reactivity of BA and Mel under various conditions indicated that a pH range of 3–7 and a temperature range of 35–65 °C could be satisfactory for the simultaneous reaction of both bases with ribose-5-phosphate (R5P). The importance of the pH to this reaction was previously recognized and confirmed by the formation of these nucleotides when carried out in separate reactions [13]. The pH necessary for the reaction of BA is above its p*K*_a_ of 3.9–4.0 [20] due to the required increase in its nucleophilicity that results from the deprotonation of the C5 carbon of BA. The exocyclic amines of Mel, on the other hand, are sufficiently nucleophilic that its reaction with the protonated aldehyde of R5P proceeds even in its neutral state. For these reasons, we focused on a solution phase of pH 5, and reaction temperatures of 20 and 33 °C.

**Figure 2:**
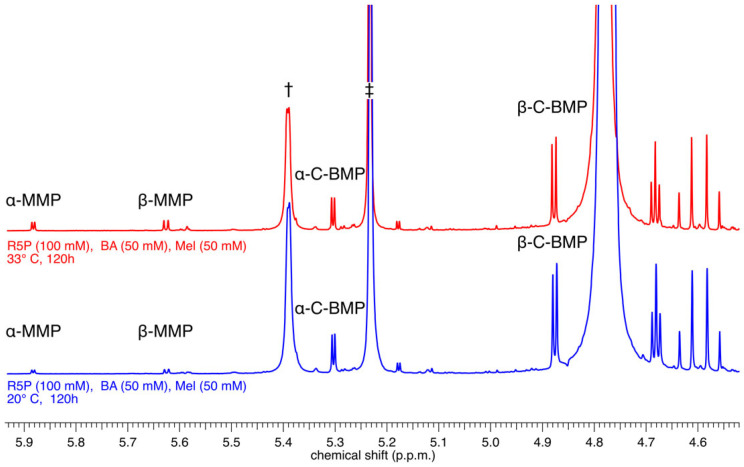
^1^H NMR analysis of the crude reaction mixture of R5P (100 mM), BA (50 mM), and Mel (50 mM) incubated at 20 °C (bottom, blue spectrum) or at 33 °C (top, red spectrum) for 120 h. Resonances corresponding to the *α*- and *β*-anomers of both MMP and BMP are observed alongside resonances for unreacted *α*- (†) and *β*-R5P (‡).

The reaction mixtures (at 20 °C and at 33 °C) containing R5P, BA, and Mel were prepared as slurries at pH 5. The solids in the stirred slurries dissolved slowly over time and became pale yellow solutions, presumably because the nucleotides being formed are more soluble than their precursor bases, an observation made previously for nucleoside-forming reactions involving the base 2,4,6-triaminopyrimidine (the pyrimidine analog of Mel) [12]. After 5 days of reaction, the solutions were worked up as described above and analyzed by ^1^H-NMR spectroscopy (Figure 2). These reactions resulted in the formation of both *α*- and *β*-anomers of BMP and MMP, which were identified by comparison with previously reported NMR spectra [13]. Specifically, the resonances associated with the two nucleotide conformers of each base were identified based on chemical shift and *J*-coupling constants that had previously been assigned to these same nucleotides and were shown to be particular to each nucleotide conformer [13]. The nucleotide products of these reactions are formed in modest but appreciable yields (calculated by integration of the ^1^H-NMR spectra). For example, the reaction carried out at 33 °C produced the *α* and *β-C* anomers of BMP in yields (based on the initial amount of nucleobase present) of 4 and 12%, respectively, while simultaneously generating the *α* and *β-C* anomers of MMP in yields of 1.2 and 1.5%, respectively (see Figure 1 for chemical structures).

**Figure 3:**
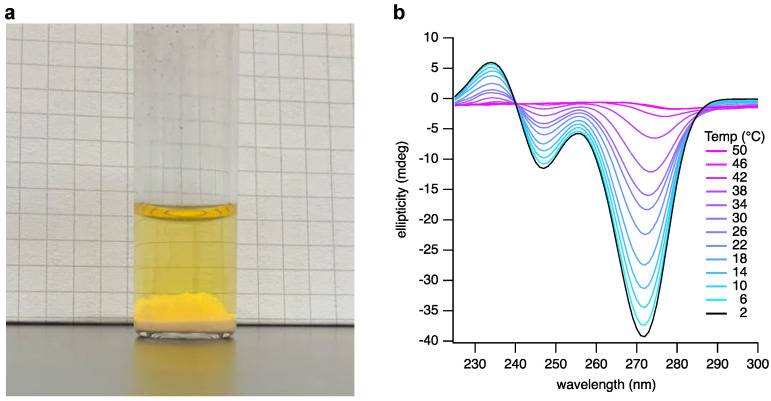
Analysis of a spontaneously assembling layer present in the crude reaction mixture of R5P, BA, and Mel. (**a**) A photograph of the crude reaction mixture after freezing and thawing. The gel phase can be seen above the thin precipitate phase and below the solution phase. (**b**) CD analysis of the assembly phase at varying temperatures, ranging from 2 to 50 °C in 4 °C increments.

Next, we observed that thawing the frozen reaction mixture gives three apparent phases: a solid phase likely to be the unreacted bases; a solution phase; and a third transitory phase suspended between the solid and the solution that was suspected to be a hydrogel (Figure 3a). We previously reported that appropriately substituted BA and Mel bases assemble as hexameric rosettes that stack in solution to generate hydrogel-forming supramolecular polymers [13]. To confirm our assumption that the third phase contained such assemblies, we isolated the gel phase and examined it by atomic force microscopy (AFM) and circular dichroism (CD) spectroscopy.

A 20 µL aliquot of the suspected gel phase was withdrawn from the thawed reaction mixture, diluted with 5 µL of 2 M NaCl, and adjusted to pH 5 to stabilize the hydrogel at the lower nucleotide concentrations that are more suitable for analysis by CD and AFM. CD spectroscopic analysis (Figure 3b) of this crude product clearly showed features characteristic of the formation of the assemblies previously observed to be formed upon mixing pure samples of BMP and MMP [13]. We also examined the temperature dependence of the CD spectrum (Figure 3b). The signals characteristic of the supramolecular assembly diminish as the temperature is increased, as expected. However, these signals clearly persist at temperatures above 30 °C.

**Figure 4:**
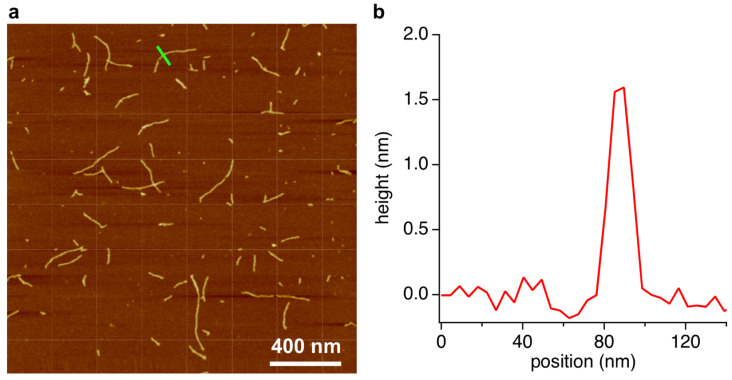
AFM analysis of the assembly phase of the crude reaction mixture of R5P, BA, and Mel. (**a**) AFM topographical image of the contents of the assembly phase after spreading on freshly-cleave mica. (**b**) A single-dimension scan across a region of this surface, denoted by the green line inscribed at the center-top of the image in panel (**a**).

The gel phase was also analyzed by AFM. We previously reported that supramolecular assemblies composed of BMP and MMP form linear structures [13]. Samples for AFM were prepared by placing 2 μL of the diluted gel phase solution on freshly cleaved mica and then spreading and drying under N_2_ flow. Excess sample was removed by rinsing with cold water, followed by drying again under N_2_ flow. The resulting images show structures with lengths ranging from 100 to 600 nm and heights of ca. 1.6 nm (Figure 4), which are consistent with the dimensions anticipated for a stacked hexad rosette assembly [11,12,13]. Hexads formed using similar compounds, i.e., a modified 2,4,6-triaminopyrimidine (isostructural with melamine) and cyanuric acid (isostructural with barbituric acid), form fibers with the same dimensions as revealed by AFM, and these assemblies were confirmed by X-ray fiber diffraction to be composed of stacked hexad assemblies as depicted in Figure 1d [21].

## 4. Discussion

The increased reactivity of the noncanonical nucleobases BA and Mel toward sugars and sugar phosphates positions them as feasible alternatives—and potential precursors—to RNA nucleobases. Their spontaneous reaction with R5P and their pairing properties both suggest that their potential as proto-nucleobases is superior to that of the canonical bases [12,13]. We recently reported that BA sequesters aldoses to form nucleosides from a dynamic, interconverting pool of ketose sugars [15]. The results reported herein extend that discovery by demonstrating the ability of both BA and Mel to form nucleotides in the same reaction, and by demonstrating these putative proto-nucleotides spontaneously form supramolecular assemblies within the crude product mixture of this one-pot reaction. While we see the organization of proto-nucleotides into supramolecular assemblies as an important step towards the prebiotic emergence of proto-RNA, it is important to note that such assemblies do not constitute a candidate for proto-RNA. Indeed, the reversible nature of these assemblies would not be able to store, transfer, or make use of nucleotide sequence information (e.g., for coding functional molecules). Rather, our goal in searching for proto-nucleotides that form supramolecular assemblies with Watson–Crick-like base pairs was to identify a possible prebiotic mechanism by which proto-nucleotides could have spontaneously organized in a manner that facilitated their covalent polymerization, a process that would produce RNA-like polymers. Hence, the next essential step in our quest to find a chemical system that provides a path to a plausible proto-RNA is to identify a prebiotic chemistry that can covalently link the proto-nucleotides that are organized within the hexad stacks. 

It is feasible that the prebiotic formation of proto-RNA provided the starting point for an evolutionary process that ultimately led to the emergence of RNA and DNA. Our demonstration that melamine and barbituric acid readily form glycosidic bonds with ribose provides a possible resolution to a major challenge to the prebiotic synthesis of RNA, that of nucleoside formation. As noted above, the propensity of melamine and barbituric acid nucleotides to form supramolecular assemblies may also provide a means to address the challenge of having a sufficiently high local nucleotide concentration to support covalent polymer formation. Despite these apparent advances, even if a one-pot synthesis can be demonstrated that gives rise to such RNA-like polymers, new questions will arise that need to be addressed, such as: How and why did a polymeric system that originated as a six-stranded assembly with three sets of two complementary sequences make the transition to a duplex system like that which is used in life today?

## Data Availability

Data is contained within the article.
